# Pueperal Paratubal Cyst Torsion and Secondary Fallopian Tube Torsion Without Ovarian Involvement: A Case Report

**DOI:** 10.7759/cureus.36540

**Published:** 2023-03-22

**Authors:** Anna Thanasa, Efthymia Thanasa, Emmanouil M Xydias, Evangelos Kamaretsos, Ioannis Paraoulakis, Apostolos C Ziogas, Vasiliki Grapsidi, Gerasimos Kontogeorgis, Ektoras-Evangelos Gerokostas, Ioannis Thanasas

**Affiliations:** 1 Department of Anatomy, Department of Health Sciences, Medical School, Aristotle University of Thessaloniki, Thessaloniki, GRC; 2 Department of Histology, Department of Health Sciences, Medical School, Aristotle University of Thessaloniki, Thessaloniki, GRC; 3 Department of Obstetrics and Gynecology, EmbryoClinic IVF, Thessaloniki, GRC; 4 Department of Obstetrics and Gynecology, General Hospital of Trikala, Trikala, GRC; 5 Department of Obstetrics and Gynecology, University of Thessaly, Larissa, GRC

**Keywords:** paratubal cyst, torsion, puerperium, ultrasound, salpingectomy, case report

## Abstract

Paratubal cyst torsion accompanied by secondary isolated fallopian tube torsion without involvement of the ipsilateral ovary is rare. A similar case occurring in the postpartum period has not been reported to date in the English literature. Our case report concerns a pregnant multiparous woman in the 40th gestational week, without regular antenatal care attendance, who was urgently admitted to the maternity ward with pushing labour pains and gave birth with vaginal delivery. A few hours later, puerperant complained of worsening severe lower abdominal pain, accompanied by nausea, dizziness and vomiting, unresponsive to analgesic medication. Based on the clinical and ultrasound findings, the diagnosis of an ovarian cyst torsion was established, and it was decided to treat the patient with surgery and in particular with laparotomy. Intraoperatively, in the left parametrium, the presence of an ovoid mass with a brownish-red hue and a smooth outer surface was detected, along which the ipsilateral fallopian tube ran, without the involvement of the ovary. Histological examination of the surgical specimen confirmed the diagnosis of isolated fallopian tubal torsion with paratubal cyst. The postoperative course was uneventful. In this paper, based on modern data, a brief literature review of this rare nosological entity is attempted, regarding the diagnostic and therapeutic approach, the immediate application of which can ensure the best prognosis.

## Introduction

The paratubal or paraovarian cyst, as otherwise referred to in the literature is of fetal origin, is estimated to originate from the Wolffian duct and was first described as a separate pathological entity by Kariminejad and Scully in 1973 [1]. The use of the terminology “paratubal” and “paraovarian” is proportional to the distance of the cyst from the fallopian tube or ovary, respectively [2]. In approximately two-thirds of the cases, their origin is estimated to be from the mesothelium of the broad ligament, while from paramesonephric or mesonephric remnants it is estimated that it develops in 30% and 2%, respectively [3]. Usually, their size does not exceed 75 mm [4], while in rare cases (13%) the maximum diameter of the cyst can exceed 150 mm [2]. Paraovarian or paratubal cysts account for approximately 5% - 20% of all adnexal masses and usually appear during the third to fourth decade of life [3]. It occurs less frequently in preadolescence and in extremely rare cases (1/1500000) it can cause isolated torsion of the ipsilateral fallopian tube [5]. Isolated torsion of the fallopian tube around its longitudinal axis is a rare cause of acute abdomen and was first described by Bland-Sutton in 1890 [6]. Similarly, malignant transformation is extremely rare [7]. It is estimated that the risk of malignancy is higher in those cases in which the size of the paratubal or paraovarian cyst is greater than 5 cm [8].

Torsion of the paratubal cyst after vaginal delivery during the immediate postpartum period has not been described in the English literature to date. Therefore, on the occasion of our patient, it is useful to emphasize the significant preoperative difficulties and the inclusion of this extremely rare nosological entity in the differential diagnosis of acute abdomen during the postpartum period.

## Case presentation

The case report concerns a 42-year-old multiparous pregnant woman, with three vaginal deliveries in her obstetric history, who, in the 40th gestational week, was urgently admitted to the maternity ward of our hospital with advanced cervical dilatation (9 cm) and pushing labour pains. She gave birth 10 minutes later by vaginal delivery to a live, full-term, male newborn weighing 3100 grams. Administration of ergometrine (0.2 mg bolus followed by 0.2 mg in drip infusion over two hours) was deemed necessary to treat postpartum hemorrhage. Obstetric history revealed incomplete antenatal care attendance of the current pregnancy. Only two visits to a gynecologist were reported, no prenatal screening was provided, nor ultrasound assessment of fetal growth during pregnancy. The personal medical history was unremarkable. Hereditary history was of no pathological significance.

**Figure 1:**
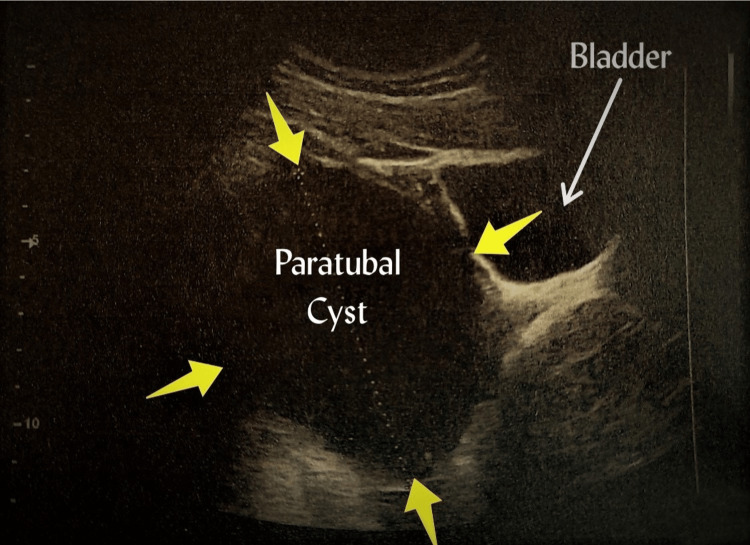
Transabdominal ultrasound imaging of a twisted paratubal cyst A unilocular, thin-walled, oval-shaped cystic formation with smooth margins in the anatomical position of the left adnexa is depicted, without simultaneous imaging of the ipsilateral ovary.

After the birth of the newborn, the urgent laboratory test revealed: hematocrit (Ht) 41.9%, hemoglobin (Hb) 13.4gr/dl, platelets (PLT) 211x103/ml, white blood cells (WBC) 16.9x103/ml, and neutrophils (NEUT) 89%. Coagulation tests were within normal range. The biochemical analysis and the urinalysis were without pathological findings. In the early hours of the morning, approximately eight hours after delivery, puerperant complained of worsening abdominal pain. She complained of severe pain in the lower abdomen, accompanied by nausea, dizziness and vomiting. The administration of analgesic drugs had no effect. From the clinical examination, no obstetric pathology was found. The peritoneal irritation signs during palpation of the left abdomen, and especially in the hypogastrium, were intense. During imaging study with the transabdominal ultrasound in the anatomical location of the left adnexa, an unilocular, thin-walled cystic mass with a regular and well-demarcated outline, with a maximum diameter of approximately 100 mm, was found (Figure 1).

**Figure 2:**
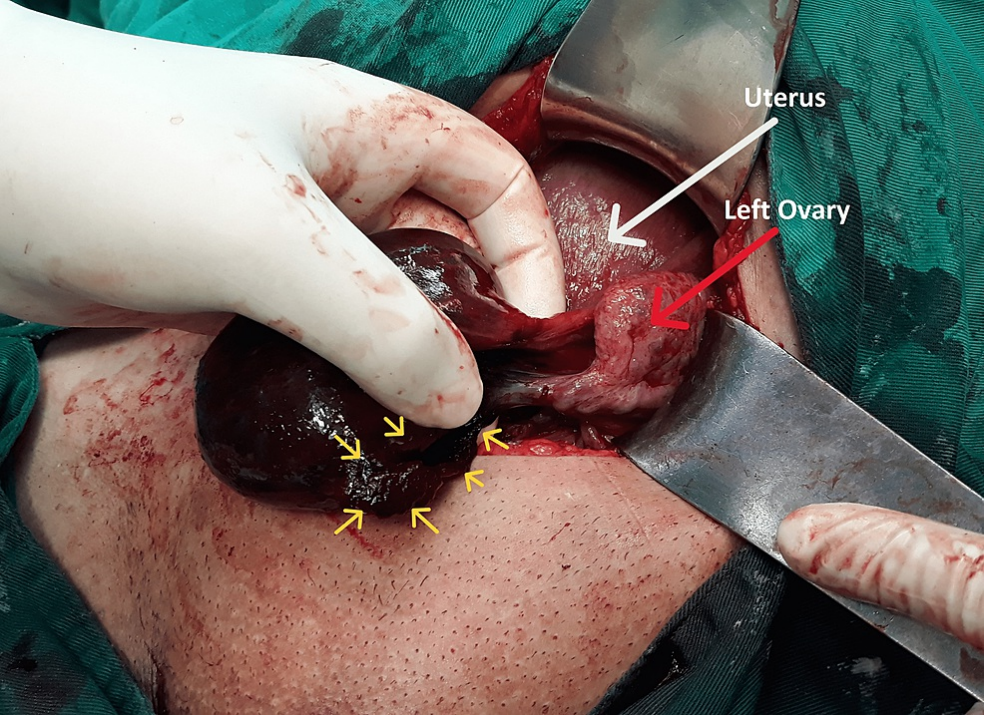
Intraoperative imaging of a twisted paratubal cyst with secondary isolated fallopian tube torsion during the immediate postpartum period The hemorrhagic infiltration and ischemic necrosis of the paratubal mass are evident, the non-participation in the lesion of the ipsilateral ovary (red arrow), as well as the characteristic running of the fallopian tube with its fimbriated end along the outer surface of the wall of the paratubal cyst (yellow arrows).

Based on the clinical and ultrasound findings, the diagnosis of ovarian cyst torsion was suspected and the open surgical treatment of the puerperant with laparotomy was decided. Laparoscopic approach was not available. Intraoperatively, the examination of adnexa, in the anatomical position of the left parametrium, revealed an ovoid mass with a maximum diameter of approximately 100 mm, with a brown-red hue and a smooth outer surface, along which the ipsilateral fallopian tube ran, without the involvement of the ovary (Figure 2). The right ovary and fallopian tube were without pathological findings.

**Figure 3:**
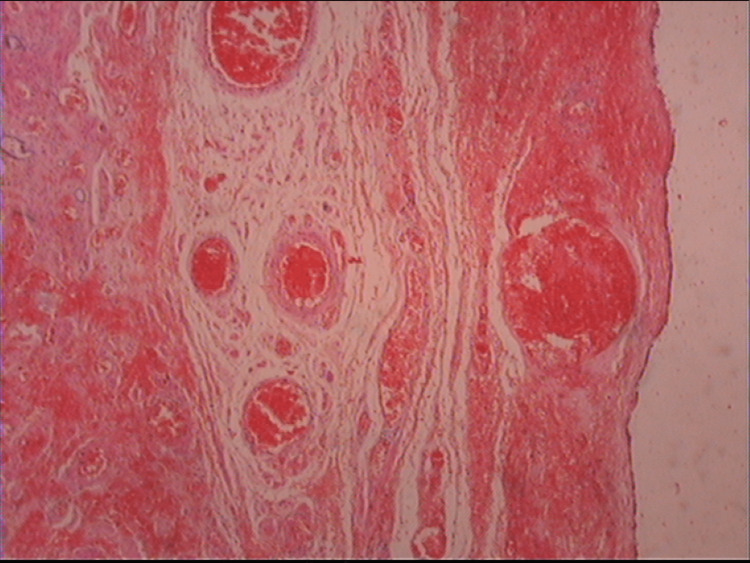
Histological image of a twisted paratubal cyst Vascular thrombosis and hemorrhagic necrosis of the cyst wall are evident.

The hemorrhagic necrotic wall of the cyst coated by cuboidal, flattened, or ciliated epithelium and the hemorrhagic necrotic fallopian tube, as revealed by microscopic examination of the surgical specimen, confirmed the diagnosis of paratubal cyst torsion with isolated fallopian tube torsion (Figure 3).

The postoperative course was uneventful. Puerperant was discharged from our clinic on the fourth postoperative day with medication and consultation for re-examination.

## Discussion

Preoperative diagnosis of paratubal cysts is a challenge in modern clinical practice. In the majority of cases, paratubal cysts are small and do not cause any clinical symptoms. Mild, dull, low-intensity abdominal pain and sudden onset of acute abdominal pain refer to large-sized lesions (cysts > 20 cm in diameter), due to pressure on the lower abdominal cavity or due to torsion of the paratubal cyst, respectively [9]. In our patient, it should be emphasized that the normal changes that occur during pregnancy and immediate postpartum period after a vaginal delivery created even more difficulties in the timely, before torsion, diagnosis of the paratubal cyst. The mild abdominal pain reported by the pregnant woman about 24 hours before her admission to the maternity ward was attributed to the common mild discomfort in the abdomen during pregnancy and especially before the onset of labor, and not to the presence of the paratubal cyst or to possible complications from it. Also, the progressively increasing abdominal pain reported by the puerperant in the immediate postpartum period was incorrectly attributed to obstetric causes rather than to the onset of fallopian tube torsion. Furthermore, in our case we did not evaluate the number of white blood cells as an inflammatory marker, since it is possible in pregnant women that the increase in the number of white blood cells can be attributed to pregnancy [10]. The leukocytosis (WBC 16.9x103/ml, NEUT 89%) found during the urgent laboratory tests of the pregnant woman admitted to the maternity ward was attributed to pregnancy and the onset of labor, and not to the onset of fallopian tube torsion with a paratubal cyst.

In many cases, abdominal pain of variable intensity can be accompanied by other symptoms related to fallopian tube torsion. Dizziness, nausea, and signs of peritoneal irritation on palpation of the abdominal wall suggest isolated fallopian tube torsion accompanied by a paratubal cyst [11]. However, the most characteristic of the accompanying symptoms is vomiting, which increases in intensity and frequency in those cases involving complete fallopian tube torsion [12]. In our patient, the repeated episodes of vomiting that occurred in the immediate postpartum period were most likely due to the isolated fallopian tube torsion with a paratubal cyst, and not to the use of semi-synthetic ergot alkaloid derivatives. A result of the misdiagnosis was the loss of the fallopian tube, with all the consequences this may have on the patient's fertility.

In contrast to clinical findings, imaging can sometimes be useful in the diagnosis of paratubal cysts. Abdominal ultrasound as a first-line test, computed tomography and magnetic resonance imaging are now considered to be of great help in the diagnostic approach to paratubal cysts, but in most cases the exact diagnosis is made during surgery [13]. On ultrasound, paratubal cysts are usually depicted as unilocular, thin-walled, round or oval-shaped cystic masses, with well-defined, smooth margins, located between the ovary and the fallopian tube, near the ipsilateral ovary, but clearly separated from it. The mobility of the cystic mass of fallopian tube and its dissociation from the ovary during pushing with the help of the transvaginal ultrasound probe are consistent characteristic sonographic features that support the diagnosis of paratubal cyst. The presence of blood flow during Doppler ultrasound cannot exclude fallopian tube torsion [11]. In our patient, the selected transabdominal ultrasound approach, the enlarged uterus that is normally observed during the immediate postpartum period and the non-completion of the ultrasound due to the intense symptoms (severe abdominal pain, persistent vomiting) were additional difficulties for the correct diagnosis of paratubal cyst torsion and its differential diagnosis from torsion of ovarian cystic mass.

Preoperative differential diagnosis between ovarian cysts and paratubal cysts remains very difficult. The incomplete description of the specific pathognomonic features on imaging when examining paraovarian cysts, especially when involved in emergency clinical situations characterized by torsion, significantly increases the risk of misdiagnosing paratubal cysts as ovarian cystic masses [14,15]. It is not surprising, in our patient, that the cystic mass in the parametrium was misdiagnosed as ovarian cyst torsion. The imaging findings of magnetic resonance imaging to date have not yielded the expected results in clinical practice. By many researchers, magnetic resonance imagining is considered preferable to computed tomography, due to the non-use of ionizing radiation, especially when it is to be used during pregnancy. Magnetic resonance imaging of unilocular or multilocular cysts with or without papillary structures from the cyst wall or with wall spread from papillary structures suggests a borderline tumor. In these cases, despite the rarity of malignant paraovarian cyst, malignant tumors and cystadenomas should be considered. Imaging of a normal ipsilateral ovary with a paratubular cyst using magnetic resonance imaging is expected to be the most important imaging feature in the diagnostic approach to paraovarian and paratubular cysts [16].

The diagnosis of paratubal cysts is easily made intraoperatively. Intraoperatively, the non-involvement of the ipsilateral ovary in the neoplasia and the characteristic running of the fallopian tube on the outer surface of the cyst are evident (Figure 2). In addition, surgical intervention (laparoscopy, laparotomy) allows radical treatment of the lesion. The laparoscopic approach with or without removal of cystic fluid seems to be preferred today in the management of paratubal cysts, provided that the surgical team is adequately trained and qualified in laparoscopic surgery. Also, it should be taken into account that the increased size of the lesion may be a limiting factor. However, in those cases where laparoscopic surgery is possible, apart from confirming the diagnosis, it is an important tool in the therapeutic management of paratubal cysts [17]. In our patient, laparoscopic access was not available. In most cases, removal of only the paratubal cyst with preservation of the ipsilateral fallopian tube is considered to be the optimal treatment, especially in young women who wish to preserve fertility. Although to date there are insufficient data available on fallopian tube function and its effect on fertility after resection of the paratubal cyst with preservation of the deformed fallopian tube, cystectomy seems to be preferred by most clinicians. Salpingectomy is indicated in cases of delayed diagnosis accompanied by ischemia and necrosis of the affected fallopian tube [18,19].

## Conclusions

Secondary isolated fallopian tube torsion accompanied by a paratubal cyst during the postpartum period is an extremely rare pathological entity. Preoperative diagnosis is a challenge in modern medical practice. Despite its uniqueness, fallopian tube torsion secondary to torsion of a paratubal cyst without ovarian involvement should be considered in the differential diagnosis of puerperal lower abdominal pain and adnexal cystic masses. During the postpartum period, abdominal pain accompanied by vomiting, before being attributed to obstetric causes, must be differentiated from adnexal torsion. Its timely treatment contributes to the preservation of the affected fallopian tube.
